# A scanning electron microscopy investigation of the precision of three orthodontic bracket slot systems

**DOI:** 10.1186/s12903-023-03841-y

**Published:** 2024-02-12

**Authors:** Mohammed Nahidh, Yassir A. Yassir, Maria Maddalena Marrapodi, Marco Di Blasio, Vincenzo Ronsivalle, Marco Cicciù, Giuseppe Minervini

**Affiliations:** 1 Department of Orthodontics, College of Dentistry, University of Baghdad, Baghdad, Iraq; 2 Department of Woman, Child and General and Specialist Surgery, University of Campania “Luigi Vanvitelli”, Naples, 80121 Italy; 3 Department of Medicine and Surgery, University Center of Dentistry, University of Parma, Parma, 43126 Italy; 4 Department of Biomedical and Surgical and Biomedical Sciences, Catania University, Catania, 95123 Italy; 5 Saveetha Dental College and Hospitals, Saveetha Institute of Medical and Technical Sciences (SIMATS), Saveetha University, Chennai, Tamil Nadu India; 6 Multidisciplinary Department of Medical-Surgical and Odontostomatological Specialties, University of Campania “Luigi Vanvitelli”, Naples, 80121 Italy

**Keywords:** Slot dimensions, Orthodontic brackets, Fixed orthodontic appliance, Orthodontics, Torque

## Abstract

**Objective:**

One of the most imprortant factors in achieving ideal teeth positions is the precision of the slot dimensions of orthodontic brackets into the archwires are inserted.This study aimed to assess the accuracy of the dimensions of orthodontic bracket slots and molar buccal tube apertures and to compare them with the specifications provided by the manufacturers.

**Method:**

A total of sixty brackets and ten molar buccal tubes with varying slot heights were examined using a scanning electron microscope from the mesial side. The dimensions and morphology of these bracket slots and buccal tubes apertures were assessed using the AutoCAD Software. A one-sample t-test was conducted to compare the measurements with the values provided by the manufacturer.

**Results:**

The findings of the present study indicated that the height of the measured bracket slots and buccal tube apertures dimensions were significantly larger than the actual dimensions and exhibiting divergent walls. On the other hand, the depth of the brackets slots showed significantly smaller values than the actual one.

**Conclusion:**

A need for careful consideration when selecting a commercially accessible brand for everyday use is essential as certain materials may not meet acceptable standards.

## Introduction

An orthodontic bracket is an attachment that can be affixed to a tooth through bonding or welded to a band. It generally comprises many components, including a base, stem, slot, tie-wings, identifying mark, and extra elements [1]. The production of metal orthodontic brackets involves casting processes, milling (such as Computer Numerical Controlled or CNC milling), and the metal injection molding method (MIM) [2].

When examining the historical development of modern orthodontic brackets, it is evident that the design of the slot has undergone significant changes. These modifications can be traced back to the introduction of the occlusal opening slot in the ribbon arch by Angle [3] and culminated in the implementation of the front opening slot in Angle’s edgewise system in 1928. The slot possessed two distinct dimensions: a vertical dimension referred to as height and a horizontal one known as depth. The initial measurements of these dimensions were recorded as 0.022 × 0.028-inch. Angle [4] implemented a non-extraction approach for treating cases involving gold archwire, which exhibited greater resilience and flexibility.

The measurements were subsequently adjusted by incorporating new measurements of 0.018 × 0.025-inch. This modification was necessitated by the high cost of gold, prompting the adoption of stainless steel alloy as a viable alternative during that period. However, the flexibility issue of the stainless steel wire posed challenges when used in the 0.022-inch slot in comparison with gold archwire [5]. Later on variations in the measurements of the bracket slot were introduced such as 0.022 × 0.030-inch and 0.018 × 0.028-inch, however; clinically, it had been found non-significant differences between 0.018 and 0.022-inch slots [6–9].

It is important to note that each type of bracket slot has advantages and disadvantages, with torque control being a significant consideration due to the size difference (play) between the archwire and the bracket slot. The issue was resolved using torquing auxiliaries, or twisting rectangular archwire to provide the necessary torque. Most of these procedures require a longer chair-side time during the wire bending process, which may cause discomfort to the patient. Additionally, the effectiveness of these methods is not guaranteed, and there is an increased risk of bracket debonding when using torqued wires [10, 11], specially when the enamel surface treated with fluoride [12] or patients consuming large amount of carbonated soft drinks [13].

As introduced by Andrews [14], the pre-adjusted brackets were designed to decrease the need for archwire bends to achieve the desired in-out, rotation, tip, and torque movements, as first proposed by Angle [4]. The archwire can acquire specific characteristics when inserted into the bracket slot. Therefore, any disparity in size between the archwire and bracket slot leads to a degree of movement or play between them. As an illustration, while retracting a maxillary incisor to mitigate an overjet, the interaction between the bracket and archwire leads to the palatal inclination of the crown, accompanied by the labial movement of the tooth’s root. The application of torque primarily in the maxillary incisors is essential for addressing the movement between the archwire and bracket, intending to achieve optimal inter-incisal angle, appropriate incisor contact, and sagittal adjustment of the dentition to attain an ideal occlusion [15, 16], both in crowded and spaced dentitions [17, 18].

Numerous studies have examined the various factors that influence torsional play. These factors encompass the materials used for brackets and archwires [19, 20], irregularities in tooth morphology, inaccuracies in bracket placement [21], and the application of bevels on archwires [12–24]. Certain researchers have made adjustments or alterations to the sizes of bracket slots to achieve the necessary torque of anterior teeth. This has been accomplished by implementing the bimetric system, bi-dimensional technique, and dual slot system [25–27].

Rubin [28] proposed the implementation of a 0.020-inch slot size to establish a standardized bracket slot size and eliminate the variability in slot sizes employed by orthodontists on a global scale. Accordingly, a slot size of 0.020-inch could serve as a viable compromise, enabling both orthodontic groups to adjust to the new dimension with minimal modifications in their selection of archwires. Additionally, orthodontic manufacturers would benefit from reduced inventory and manufacturing equipment requirements. Consequently, these potential savings could lead to decreased prices. The slot size mentioned was utilized exclusively in two in-vitro studies [29, 30].

Orthodontists must possess comprehensive knowledge regarding brackets manufacturing techniques and slot dimensions accuracy. This is because manufacturers do not provide information regarding the method of measuring bracket slots or the specified tolerance limits for slot dimensions in their product catalogs. Consequently, the torque required for effective treatment may be compromised [31].

The introduction of orthodontic product standards in 1998 and 2000 encompassed the description of nominal dimensions for brackets and wires and the establishment of tolerance limitations [32, 33]. The ISO 27,020 [34] specification was released to establish the tolerance limits and measuring procedure. DIN 13971-2 [33] specified an admissible tolerance limit of 0.04 mm; however, the ISO 27,020 [34] specification opted for a tolerance limit of 0.01 mm.

The objective of the current investigation was to conduct a comparative analysis of the three bracket slot dimensions (0.018, 0.020, and 0.022-inch) in relation to the specifications provided by the manufacturer. The null hypothesis posited that no statistically significant difference exists between the measured and reported bracket slot and molar buccal tube aperture dimensions.

## Materials and methods

### Sample

Based on the bracket slot dimensions, three MBT systems were selected from Hangzhou Xingchen 3B Dental Instruments and Materials Co., China. They were distributed as followed:

Group A: comprised Diamond Star brackets with slot dimensions of 0.018 × 0.028-inch for the anterior teeth and 0.022 × 0.028-inch for the posterior teeth including the molar tubes (Hybrid system), identified by a Lot number “BR19120531”. The body of the brackets was produced using the MIM technology, while the slots were created by CNC milling, with a specified tolerance limit of + 0.06 mm.

Group B: included Trumpet brackets and tubes measuring 0.020 × 0.028-inch, which were utilized for all teeth and assigned with a specific Lot number “BR19120530”. The brackets were produced using MIM technology, with a tolerance limit of + 0.06 mm.

Group C: comprised Diamond Star brackets and tubes of 0.022 × 0.028-inch for all teeth, each with a specific Lot number “BR19120529”. The manufacturing procedure closely resembles the brackets utilized by group A.

The sample size was determined according to the previous studies [35, 36], so a total of 60 orthodontic brackets were chosen for analysis, consisting of 20 right maxillary brackets from each of the following tooth groups: central incisors, lateral incisors, canines, and second premolars (5 form each). Additionally, ten molar buccal tubes were included in the study, with five tubes from group B measuring 0.020-inch in height and five tubes from group C measuring 0.022-inch in height. All of these tubes were manufactured using MIM technology.

### Methods

Scanning electron microscopy was employed to examine the brackets and tubes from the mesial side. The specific microscope used for this analysis was the AxiaChemi SEM, manufactured by Thermo Fisher Scientific Inc., Netherlands.

According to the method described by Brown et al. [37], the brackets were positioned and fixed on a microscope table using special adhesive tape so as they were orientated vertically with the slots aligned parallel to the line of view of the measuring microscope. The resulting image was then digitally taken.

The heights of the slots were measured using AutoCAD software. The measurements were taken at a distance of 100 μm (0.1 mm) from the slot deepest point and outside border. This was done to minimize any bias caused by the roundness of the slot angles, as suggested by previous studies [37, 38].

**Fig. 1:**
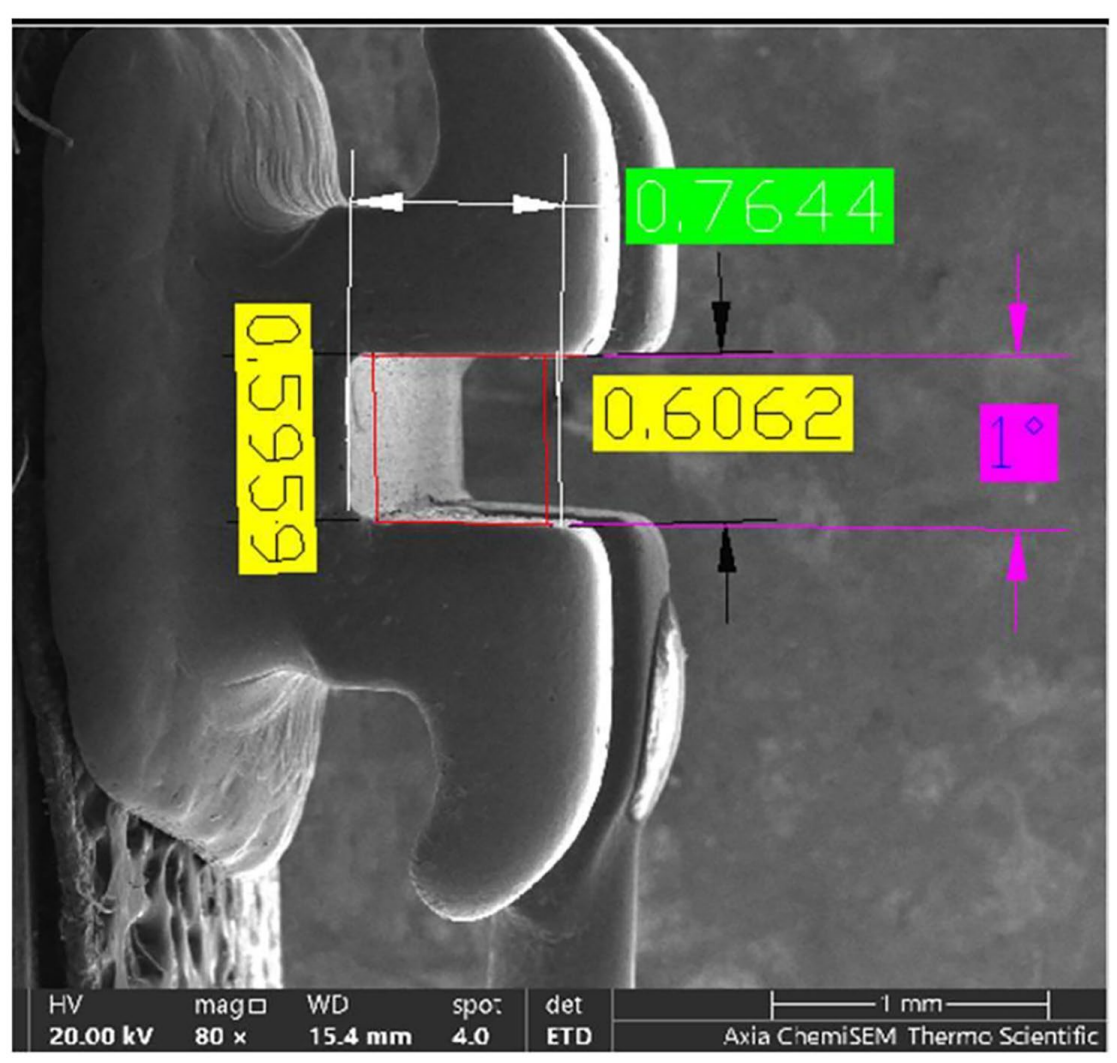
Measuring the dimensions and parallelism of the brackets slot (The height at the base and the face are in yellow color, the depth is in green color and the parallelism is in pink color)

The measurement of depth was conducted by determining the distance from the deepest point to the outer border of the slot, as described by El-Angabawy [39]. The measurement of wall parallelism involved determining the angle between the slot upper and lower internal walls, as depicted in Fig. 1.

**Fig. 2:**
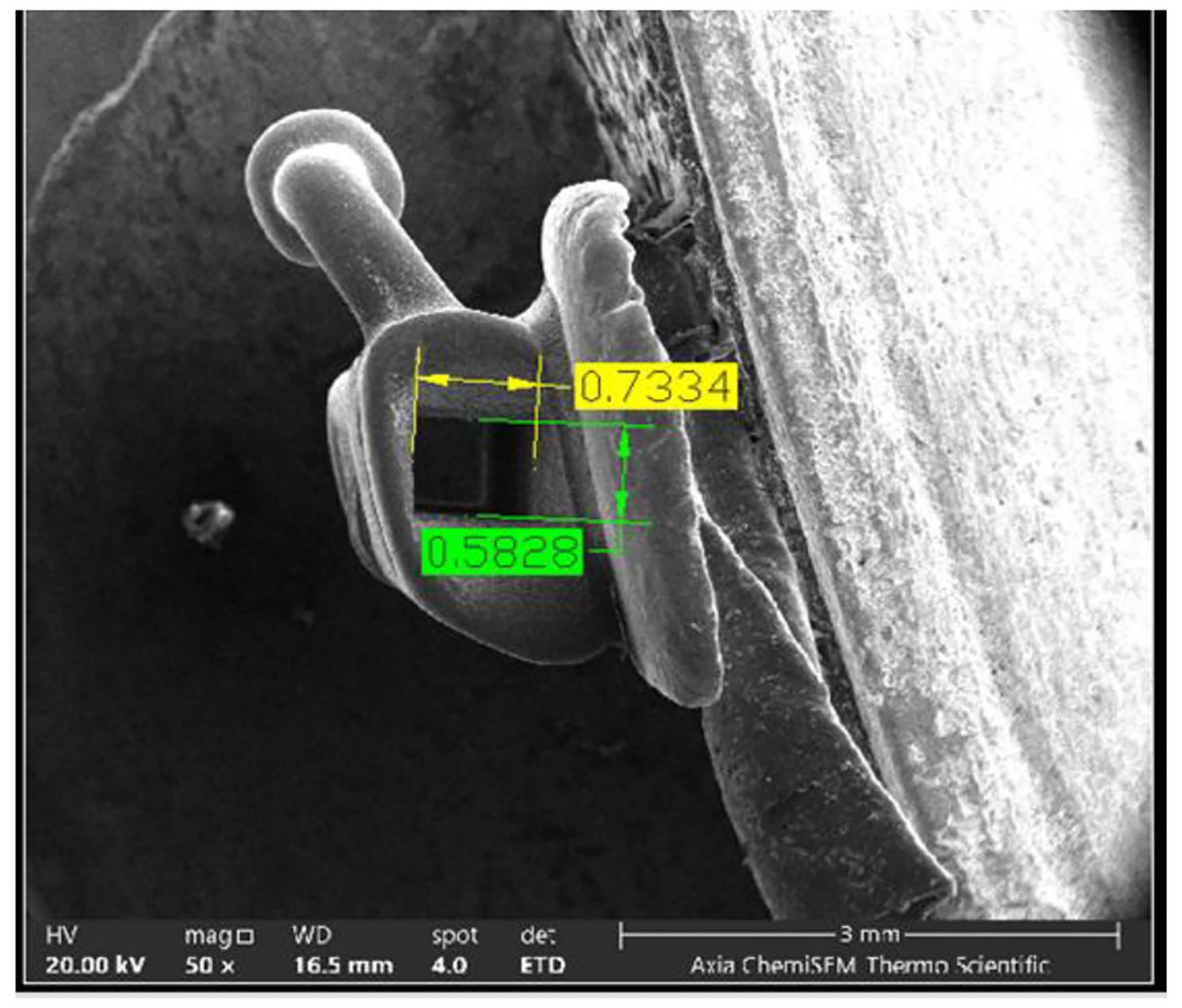
Measuring the dimensions of the front aperture of the molar buccal tube (the height is in green color and the width is in yellow color)

In order to evaluate the accuracy of the front aperture dimensions of the buccal molar tubes, a total of 10 tubes were chosen, consisting of 5 right maxillary first molar buccal tubes from groups B and C. These selected tubes were positioned like the bracket and subjected to SEM analysis from the mesial side. The measurements of the heights and widths were conducted using AutoCAD software, following the methodology proposed by Al-Zubaidi and Alhuwaizi [40], as depicted in Fig. 2.

### Statistical analysis

The data analysis was conducted utilizing SPSS (Statistical Package for Social Sciences) software developed by IBM SPSS Inc. (Chicago, IL, USA). Descriptive statistics were computed, and a one-sample t-test was employed to compare the actual slot dimensions with the stated dimensions. Intraclass correlation coefficient was used to check the inter and intr-examiner reliability. The significance level was established at 0.05.

## Results

Firstly, inter- and intra-examiner reliabilities were tested using intraclass correlation coefficient (ICC). The bracket dimensions and geometry of five samples measured twice with a month interval. An ICC showed high reliability for inter- and intra-examiner measurements.

The measurements of the slots of various brackets utilized in this investigation and the morphology of the brackets were provided in Tables 1, 2 and 3. The results of the one-sample t-test revealed that the mean values of the slot height in all groups were greater significantly than the actual one, the reverse is true for the depth. The heights of the slots exhibited an upward trend from the base to the face, characterized by a slightly divergent geometric configuration.

**Table 1:** Descriptive statistics and comparison between the measured and stated dimensions in group A (hybrid system)

Brackets	Dimensions	Descriptive statistics				Difference		
		Mean	S.D.	Min.	Max.	% of difference	t-test	*p*-value
**Central** **incisor**	**Height [Base]**	0.0198	0.00014	0.0196	0.0200	0.18	28.46	0.0001
	**Height [Face]**	0.0202	0.00013	0.0201	0.0204	0.22	38.073	0.0001
	**Depth**	0.0262	0.00011	0.0260	0.0263	-0.18	-36.085	0.0001
	**Angle**	1.4000	0.54772	1	2	0.014	5.715	0.005
**Lateral** **incisor**	**Height [Base]**	0.0196	0.00010	0.0195	0.0197	0.16	35.777	0.0001
	**Height [Face]**	0.0204	0.00047	0.0201	0.0212	0.24	11.424	0.0001
	**Depth**	0.0236	0.00008	0.0235	0.0237	-0.44	-117.06	0.0001
	**Angle**	1.6000	0.54772	1	2	0.016	6.532	0.003
**Canine**	**Height [Base]**	0.0198	0.00008	0.0197	0.0199	0.18	48.642	0.0001
	**Height [Face]**	0.0203	0.00010	0.0202	0.0204	0.23	51.34	0.0001
	**Depth**	0.0272	0.00008	0.0271	0.0273	-0.08	-20.846	0.0001
	**Angle**	1.4000	0.89443	0	2	0.014	3.5	0.025
**Second** **premolar**	**Height [Base]**	0.0237	0.0001	0.0236	0.0238	0.17	53.759	0.0001
	**Height [Face]**	0.0243	0.0001	0.0241	0.0244	0.23	39.102	0.0001
	**Depth**	0.0234	0.0001	0.0233	0.0234	-0.46	-189.427	0.0001
	**Angle**	2.8000	0.4472	2	3	0.028	14	0.0001

The depth and the height at the base and the face were measured in inch. The angle between the upper and lower walls was measured in degree

**Table 2:** Descriptive statistics and comparison between the measured and stated dimensions in group B (0.020-inch slot)

Brackets	Dimensions	Descriptive statistics				Difference		
		Mean	S.D.	Min.	Max.	% of difference	t-test	*p*-value
**Central** **incisor**	**Height [Base]**	0.0205	0.0001	0.0204	0.0206	0.05	15.811	0.0001
	**Height [Face]**	0.0209	0.0002	0.0207	0.0213	0.09	8.581	0.001
	**Depth**	0.0239	0.0008	0.0229	0.0249	-0.41	-11.327	0.0001
	**Angle**	2	0.7071	1	3	0.02	6.325	0.003
**Lateral** **incisor**	**Height [Base]**	0.0218	0.0006	0.0212	0.0226	0.18	6.903	0.002
	**Height [Face]**	0.0221	0.0004	0.0213	0.0223	0.21	10.624	0.0001
	**Depth**	0.0232	0.0014	0.0214	0.0245	-0.48	-7.883	0.001
	**Angle**	1.6000	0.5477	1	2	0.016	6.532	0.003
**Canine**	**Height [Base]**	0.0209	0.0005	0.0204	0.0215	0.09	3.553	0.024
	**Height [Face]**	0.0218	0.0005	0.0211	0.0222	0.18	8.009	0.001
	**Depth**	0.0291	0.0006	0.0284	0.0296	0.11	4.203	0.014
	**Angle**	1.8000	0.4472	1	2	0.018	9	0.001
**Second** **premolar**	**Height [Base]**	0.0216	0.0001	0.0215	0.0217	0.16	50.596	0.0001
	**Height [Face]**	0.0224	0.0001	0.0223	0.0225	0.24	75.895	0.0001
	**Depth**	0.0260	0.0005	0.0256	0.0268	-0.2	-9.378	0.001
	**Angle**	2.6000	0.5477	2	3	0.026	10.614	0.0001

The depth and the height at the base and the face were measured in inch. The angle between the upper and lower walls was measured in degree

**Table 3:** Descriptive statistics and comparison between the measured and stated dimensions in group C (0.022-inch slot)

Brackets	Dimensions	Descriptive statistics				Difference		
		Mean	S.D.	Min.	Max.	% of difference	t-test	*p*-value
**Central** **incisor**	**Height [Base]**	0.0219	0.0002	0.0217	0.0221	-0.01	-1.414	0.23
	**Height [Face]**	0.0232	0.0001	0.0231	0.0233	0.12	32.606	0.0001
	**Depth**	0.0252	0.0006	0.0245	0.0259	-0.28	-11.005	0.0001
	**Angle**	1.6000	0.5477	1	2	0.016	6.532	0.003
**Lateral** **incisor**	**Height [Base]**	0.0225	0.0001	0.0225	0.0226	0.05	22.045	0.0001
	**Height [Face]**	0.0229	0.0001	0.0228	0.0230	0.09	24.588	0.0001
	**Depth**	0.0245	0.0001	0.0243	0.0247	-0.35	-52.463	0.0001
	**Angle**	1.2000	0.4472	1	2	0.012	6	0.004
**Canine**	**Height [Base]**	0.0231	0.00004	0.0231	0.0232	0.11	56	0.0001
	**Height [Face]**	0.0237	0.0001	0.0237	0.0238	0.17	71.035	0.0001
	**Depth**	0.0281	0.00004	0.0281	0.0282	0.01	6	0.004
	**Angle**	2.6000	0.5477	2	3	0.026	10.614	0.0001
**Second** **premolar**	**Height [Base]**	0.0237	0.0001	0.0236	0.0238	0.17	53.759	0.0001
	**Height [Face]**	0.0243	0.0001	0.0241	0.0244	0.23	39.102	0.0001
	**Depth**	0.0234	0.0001	0.0233	0.0234	-0.46	-189.427	0.0001
	**Angle**	2.8000	0.4472	2	3	0.028	14	0.0001

The depth and the height at the base and the face were measured in inch. The angle between the upper and lower walls was measured in degree

About the measurements of the buccal tubes in groups B and C, it was seen that both the height and width of these tubes were found to be significatly larger than the stated one (Table 4).

**Table 4:** Descriptive statistics and comparison between the measured and stated dimensions for the buccal tubes in groups B and C

Groups	Dimensions [Inch]	Descriptive statistics				Difference		
		Mean	S.D.	Min.	Max.	% of difference	t-test	*p*-value
**B**	**Height**	0.0208	0.00002	0.0207	0.0208	0.08	77.2110	0.0001
	**Width**	0.0284	0.0001	0.0282	0.0285	0.04	6.0310	0.0040
**C**	**Height**	0.0237	0.0003	0.0235	0.0241	0.17	14.0960	0.0001
	**Width**	0.0286	0.0002	0.0283	0.0288	0.06	6.2580	0.0030

## Discussion

This study aimed to assess the accuracy of the slot dimensions in three different orthodontic bracket systems. Three systems were evaluated in this stuy. In group A, the slot height for anterior teeth was 0.018-inch, while for posterior teeth, it was 0.022-inch. The slot heights for groups B and C were measured to be 0.020 and 0.022-inch, respectively. This study used two different dimensions of buccal molar tubes, precisely 0.020 and 0.022-inch sizes, denoted as groups B and C, respectively.

The manufacturer has implemented a tolerance limit of 0.06 mm (equivalent to 0.0024 inch). Based on the specifications outlined in DIN 13971-2 [33] and ISO 27,020 [34], it is recommended that the tolerance for industrial slot height be set at 0.04 mm and 0.01 mm, respectively. This indicates that the tolerance limit established by the company somewhat exceeds that of DIN 13971-2 [33].

In order to mitigate potential bias arising from the roundness of the slot angles, a distance of 100 μm (0.1 mm) was maintained between the deepest point and outside border of the slot, as recommended by previous studies [37, 38]. The authors in the majority of other studies did not emphasize this particular topic.

The measurement of the slot in earlier research was conducted using various devices. These options include their own set of advatages and disadvantages. The current investigation employed a scanning electron microscope due to its ability to offer substantial magnification and provide detailed information regarding the topography, morphology, and composition of the metal samples, which encompassed brackets, molar tubes, and archwires [41].

The null hypothesis, which posits no difference in the dimensions of bracket slots and buccal tubes compared to the stated dimensions, was rejected based on the findings of this study. The results indicate that the heights of all bracket slots were larger than the stated dimensions but still within the acceptable range specified by the company. This aligns with recent studies, which found that most bracket slot heights exceed the manufacturers’ specifications [42–44].

According to Cash et al. [45], significant discrepancies were observed between the measured and reported values of slot height, with the majority of bracket slots being larger than indicated. These differences ranged from 2.26 to 24%. The percentages of difference in the present study were lower than the previous study. The depth of the slot typically exhibits smaller values based on the constraints imposed by the upper and lower slot walls, as the torque is primarily concentrated in the face rather than the base of the bracket. The molar buccal tubes had diameters that exceeded the specified measurements, aligning with the findings of Al-Zubaidi and Alhuwaizi [40].

A limited number of investigations have documented the presence of a bracket slot that is insufficiently small. While the standard allows for undersized slots within the specified tolerance limits, it is essential to note that these holes may impede the appropriate insertion of a wire with a cross-sectional area corresponding to the nominal slot size [46]. In the clinical setting, it is observed that the full-size archwire has the potential to be accommodated in smaller slots. This is attributed to the fact that contemporary archwires are frequently smaller than their designated specifications [47]. The observed variation can be ascribed to multiple factors, such as the specific bracket manufacturing technique employed (e.g., casting, milling, and MIM), the measuring apparatus utilized, the position of measurement within the slot, the specific side of measurement (mesial, distal, or both), and even potential variations among batches of the same brand.

Due to their extended production cycles and reduced cost-effectiveness, casting and milling are less often employed manufacturing techniques for bracket bodies compared to the more prevalent method of MIM [48]. According to Khan [2], several bracket systems use MIM to produce the bracket body, while employing machining or milling techniques to create the bracket slot. The current study employed the MIM technology to produce the brackets in group B, but for groups A and C, the bodies were fabricated using MIM, while the slots were created using the CNC milling technique.

In accordance with the results of the current investigation, Tepedino et al. [47] discovered that bracket slot heights consistently exceed the required dimensions, irrespective of the production techniques employed. In addition, Park et al. [49] conducted a study on the slot size and wall parallelism of metal brackets manufactured using MIM and CNC milling and the results indicated that the entire sample of brackets exhibited significant larger slot sizes. Furthermore, out of the seven systems analyzed, only one demonstrated parallel walls, while the remaining systems exhibited divergent walls. The researchers could not establish that CNC milling exhibited superior precision as a production technology compared to MIM. Additionally, Martínez et al. [38] evaluated several orthodontic brackets manufactured using MIM technology and found that four of the tested bracket systems exhibited a mean slot height value that exceeded the tolerance limitations specified in the ISO 27,020 standard.

According to Eliades et al. [50], using MIM as a production method for brackets is deemed the most economically efficient. However, it should be noted that this approach necessitates the utilization of a mold that is 18 to 20% larger to accommodate the shrinkage that occurs during the sintering process. The extent of shrinkage can be influenced by several factors, including the alloy composition, kind of powder used, de-binding process, rate of sintering heat, and duration of sintering hold. These variables might affect the ultimate dimensions of the material. The occurrence of large slots can be attributed to either a failure in accurately calculating the shrinkage during the sintering process or a lack of precision in controlling the final polishing stage. Furthermore, using quality control measures utilizing gauges enables the expedited identification and rejection of small brackets compared to larger brackets [2, 50].

The orthodontic brackets produced by casting and milling are subject to the influence of shrinkage, resulting in imperfections such as grooves and striations on the surfaces of the porous slot walls. In order to address manufacturing defects and ensure that they do not impede the functionality of archwires, manufacturers intentionally increase the size of the slot and create beveled edges on the archwires [15]. In the current investigation, the heights of the slots have been increased.

Dimensional imprecision can arise due to several factors in the production process, and if left unaddressed, interbatch differences may occur. The correctness of several batches of brackets was investigated solely by Martínez et al. [38]. The findings of this inquiry indicate significant variability across batches in six of the twelve systems examined. This variability can be attributed to insufficient validation of the manufacturing process or the lack of an effective quality control mechanism.

The ultimate goals of process validation operations encompass achieving consistency and uniformity both within a single batch and across multiple batches. According to Schmidli and Grize [51], a verified process possesses sufficient safeguards against potential sources of unpredictability that could negatively impact industrial output. In order to exclude the influence of this variable, the batches were standardized for each group of appliances in the current investigation.

Ideally, the upper and lower walls of the slot should exhibit a high degree of similarity, being perpendicular to the bottom, possessing a smooth surface, and being free from any irregularities or impurities [37]. The present study resembles previous investigations that have also identified the lack of parallelism in the slot wall surfaces. In their study, Cash et al. [45] observed the presence of parallel walls in just three out of the eleven systems they investigated. Conversely, the majority of the systems they analyzed displayed converging walls. In contrast, Lefebvre et al. [52] documented divergent walls in a significant proportion of their respective samples, precisely 84–85% and 100%. In a study conducted by Park et al. [49], it was observed that out of the eight bracket systems examined, diverging walls were present in seven systems, while only one exhibited parallel walls. Although the brackets tested in the studies mentioned above were manufactured using MIM and milling procedures similar to those employed in the present investigation, no conclusive evidence regarding their geometric accuracy was found.

One drawback of the current study is that the measurement of bracket slots was limited to the mesial side only, with no corresponding measurements made from the distal side of the brackets. However, it is worth noting that previous studies have indicated that any potential differences between the two sides were not statistically significant [53–64].

## Conclusions

The findings of this study suggest that height of the orthodontic bracket slots exhibited significant oversize dimensions with diverged slots, the same is true for the dimension of the molar buccal tubes apertures. Moreover, it is recommended that future research and development efforts in the industry focus on incorporating innovative technologies into the production process of brackets to eliminate this particular drawback and using biomimetic hydroxyapatite incorporated in the composite adhesive or coating the brackets with nanoparticles with anti-bacterial effect to reduce the incidence of lesions on the hard tissues of the tooth.

## Data Availability

The data will be available on reasonable request from the corresponding author.

## References

[1] Daskalogiannakis J (2000). Glossary of orthodontic terms.

[2] Khan H (2015). Orthodontic brackets selection, placement and debonding.

[3] Angle EH (1916). Some new forms of orthodontic mechanism and the reason for their introduction. Dent Cosmos.

[4] Angle EH (1928). The latest and best in orthodontic mechanism. Dent Cosmos.

[5] Steiner CC (1953). Power storage and delivery in orthodontic appliances. Am J Orthod.

[6] Yassir YA, El-Angbawi AM, McIntyre GT, Revie GF, Bearn DR (2018). A randomized clinical trial of the effectiveness of 0.018-inch and 0.022-inch slot orthodontic bracket systems: part 1-duration of treatment. Eur J Orthod.

[7] Yassir YA, El-Angbawi AM, McIntyre GT, Revie GF, Bearn DR (2018). A randomized clinical trial of the effectiveness of 0.018-inch and 0.022-inch slot orthodontic bracket systems: part 2—quality of treatment. Eur J Orthod.

[8] Mastrangelo F, Nargi E, Carone L, Dolci M, Caciagli F, Ciccarelli R (2008). Tridimensional Response of human Dental Follicular Stem cells onto a Synthetic Hydroxyapatite Scaffold. J Health Sci.

[9] Yassir YA, McIntyre GT, El-Angbawi AM, Bearn DR (2019). Does anchorage loss differ with 0.018-inch and 0.022-inch slot bracket systems?. Angle Orthod.

[10] Mastrangelo F, Piccirilli M, Dolci M, Teté S, Speranza L, Patruno A (2005). Vascular endothelial growth factor (VEGF) in human tooth germ Center. Int J Immunopathol Pharmacol.

[11] Pancherz H, Löffler A, Obijou C (2001). Efficiency of root torquing auxiliaries. Clin Orthod Res.

[12] Lanteri V, Segù M, Doldi J, Butera A (2014). Pre-bonding prophylaxis and brackets detachment: an experimental comparison of different methods. Inter J Clin Dent.

[13] Al-Hussain ZAA, Nahidh M (2021). Carbonated soft drinks and orthodontics: review of literature. Turk J Orthod.

[14] Andrews LF (1976). The straight-wire appliance: origin, controversy, commentary. J Clin Orthod.

[15] Kusy RP, Whitley JQ (1999). Assessment of second order clearances between orthodontic archwires and bracket slots via the critical contact angle for binding. Angle Orthod.

[16] Archambault A, Lacoursiere R, Badawi H, Major PW, Carey J, Flores-Mir C (2010). Torque expression in stainless steel orthodontic brackets: a systematic review. Angle Orthod.

[17] Dos Santos CCO, da Rosa Moreira Bastos RT, Bellini-Pereira SA, Garib D, Normando D (2023). Spontaneous changes in mandibular incisor crowding from mixed to permanent dentition: a systematic review. Prog Orthod.

[18] Patano A, Malcangi G, Inchingolo AD (2023). Mandibular crowding: diagnosis and management-A scoping review. J Pers Med.

[19] Fischer-Brandies H, Orthuber W, Es-Souni M, Meyer S (2000). Torque transmission between square wire and bracket as a function of measurement, form and hardness parameters. J Orofac Orthop.

[20] Gioka C, Eliades T (2004). Materials-induced variations in the torque expression of preadjusted appliances. Am J Orthod Dentofacial Orthop.

[21] Miethke RR, Melsen B (1999). Effect of variation in tooth morphology and bracket position on first and third order correction with preadjusted appliances. Am J Orthod Dentofacial Orthop.

[22] Sebanc J, Brantley WA, Pincsak JJ, Conover JP (1984). Variability of effective torque as a function of edge bevel on orthodontic arch wires. Am J Orthod.

[23] Meling TR, Odegaard J, Meling E (1993). A theoretical evaluation of the influence of variation in bracket slot height and wire rounding on the amount of torsional play between bracket and wire. Kieferorthop Mittlg.

[24] Meling TR, Odegaard J (1997). On the mechanical properties of square and rectangular stainless steel wires tested in torsion. Am J Orthod Dentofacial Orthop.

[25] Schudy FF, Schudy GF (1975). The bimetric system. Am J Orthod.

[26] Gianelly AA. Bidimensional technique: theory and practice. 1st ed. Fenwyn Press; 2000.

[27] Rinchuse DJ, Rinchuse DJ (2011). Modification of the bidimensional system. Orthodontics: The Art & Practice of Dentofacial Enhancement.

[28] Rubin RM (2001). Letters from our readers: RE: a Plea for Agreement. Angle Orthod.

[29] Al-Jumaili KA, Ali AI, Shindala HM (2014). Effect of bracket’s slot size on canine position and space closure rate (a typodont study). Al-Rafidain Dent J.

[30] Kim JY, Yu WJ, Koteswaracc PN, Kyung HM (2017). Effects of bracket slot size during en-masse retraction of the six maxillary anterior teeth using an induction-heating typodont simulation system. Korean J Orthod.

[31] Meling T, Odegaard J, Holte K, Meling E, Segner D (1998). A formula for the displacement of an arch wire when subjected to a second-order couple. Am J Orthod Dentofacial Orthop.

[32] Deutsches Institut fu¨ r Normung e.V. DIN 13971: 1998-01 Zahnheilkunde- Kieferorthopa¨dische Dra¨ hte.

[33] Deutsches Institut. fu¨ r Normung e.V. DIN 13971-2: 2000-01 Kieferorthopa¨dische Produkte.

[34] ISO 27020. Dentistry-Brackets and tubes for use in orthodontics. https://www.iso.org/standard/72549.html. 2019.

[35] Daga PN, Karandikar GR, Patni V, Karandikar AG, Doshi S (2017). A comparative evaluation of accuracy of Mclaughlin Bennet Trevisi prescription of six commercially available orthodontic metal brackets: an in vitro study. J Indian Orthod Soc.

[36] Radhakrishnan PD, Sapna Varma NK, Ajith VV (2017). Assessment of bracket surface morphology and dimensional change. Contemp Clin Dent.

[37] Brown P, Wagner W, Choi H (2015). Orthodontic bracket slot dimensions as measured from entire bracket series. Angle Orthod.

[38] Martínez LB, Garcovich D, Pamplona PE, Martín MAA, Lorenzo AA (2021). Compliance with the ISO 27020: 2019 norm of a sample of currently available preadjusted orthodontic bracket systems. Are the actual dimensions as expected?. Head & Face Med.

[39] El-Angbawi AMF. Is the 0.018-inch or the 0.022-inch bracket slot system more effective for the levelling and alignment stage of orthodontic treatment? A Ph.D. Thesis, University of Dundee, 2013.

[40] Al-Zubaidi HJ, Alhuwaizi AF (2018). Molar buccal tubes front and back openings dimensions and torsional play. J Baghdad Coll Dentistry.

[41] Choudhary P, Priyanka (2017). Scanning electron microscope: advantages and disadvantages in imaging components. Int J Current Microbiol Applied Sci.

[42] Nahidh M, Yassir YA (2023). Evaluating orthodontic bracket slot dimensions and morphology: a narrative review. J Orthod Sci.

[43] Garrett A, Alghilan MA, Ash S, Awawdeh M, Singh P (2023). An evaluation of the accuracy and precision of ceramic orthodontic bracket slot dimensions utilizing micro-computed tomography (Micro-CT). Tomography.

[44] Manzoor Z, Nagar S, Kumar A, Singh M, Agrawal N, Thareja V (2023). In vitro comparative evaluation of slot size and in-built torque of different passive self-ligating bracket systems: a stereo-microscopic study. Eur Chem Bull.

[45] Cash AC, Good SA, Curtis RV, McDonald F (2004). An evaluation of slot size in orthodontic brackets- are standards as expected?. Angle Orthod.

[46] Awasthi E, Sharma N, Shrivastav S, Kamble RH (2015). Evaluation and comparison of various prescription specifications and slot distortion of pre-adjusted edgewise brackets manufactured by different companies available in India. Int J Cur Res Rev.

[47] Tepedino M, Paiella G, Potrubacz MI, Monaco A, Gatto R, Chimenti C (2020). Dimensional variability of orthodontic slots and archwires: an analysis of torque expression and clinical implications. Prog Orthod.

[48] Supriadi S, Sitanggang TW, Bambang Irawan S, Suharno B, Kiswanto G, Prasetyadi T (2015). Orthodontic bracket fabrication using the investment casting process. Int J Technol.

[49] Park JS, Song IT, Bae JH, Gil SM, Kang KH (2020). Comparison of slot sizes and parallelism of metal brackets manufactured through metal injection molding and computerized numerical control. Korean J Orthod.

[50] Eliades T, Zinelis S, Bourauel C, Eliades G (2008). Manufacturing of orthodontic brackets: a review of metallurgical perspectives and applications. Recent Pat Mater Sci.

[51] Schmidli H, Grize Y-L (1997). Quantification of batch homogeneity. Qual Engeneering.

[52] Lefebvre C, Saadaoui H, Olive J-M, Renaudin S, Jordana F (2019). Variability of slot size in orthodontic brackets. Clin Exp Dent Res.

[53] Khan T, Khan H, Mohsin S, Ahmad F, Saeed MQ (2018). Manufacturer tolerance in mesial and distal slot height of 0.022-inch maxillary lateral incisor brackets. Pakistan Orthod J.

[54] Vozzo LM, Azevedo L, Fernandes JCH, Fonseca P, Araújo F, Teixeira W, Fernandes GVO, Correia A. The success and complications of complete-arch implant-supported fixed monolithic zirconia restorations: a systematic review. Prosthesis. 2023;5(2):425–36. 10.3390/prosthesis5020029.

[55] Chhikara K, Gupta S, Bose D, Kataria C, Chanda A. Development and trial of a multipurpose customized orthosis for activities of daily living in patients with spinal cord injury. Prosthesis. 2023;5(2):467–79. 10.3390/prosthesis5020032.

[56] Iacono R, Mayer Y, Marenzi G, Ferreira BV, Pires GE, Migliorati M, Bagnasco F. Clinical radiological and aesthetic outcomes after placement of a bioactive-surfaced implant with immediate or delayed loading in the anterior maxilla: 1-year retrospective follow-up study. Prosthesis. 2023;5(3):610–21. 10.3390/prosthesis5030043.

[57] Di Francesco F, Lanza A, Di Blasio M, Vaienti B, Cafferata EA, Cervino G, Cicciù M, Minervini G. Application of botulinum toxin in temporomandibular disorders: a systematic review of randomized controlled trials (RCTs). Appl Sci. 2022;12(23):12409. 10.3390/app122312409.

[58] Minervini G, D’Amico D, Cicciù M, Fiorillo L. Temporomandibular joint disk displacement: etiology diagnosis imaging and therapeutic approaches. J Craniofac Surg. 2023;34(3):1115–21. 10.1097/SCS.0000000000009103. 10.1097/SCS.0000000000009103 36730822

[59] Crescente G, Minervini G, Spagnuolo C, Moccia S. Cannabis bioactive compound-based formulations: new perspectives for the management of orofacial pain. Molecules. 2023:28(1)106. 10.3390/molecules28010106. 10.3390/molecules28010106 PMC9822121 36615298

[60] Minervini G, Del Mondo D, Russo D, Cervino G, D’Amico C, Fiorillo L. Stem cells in temporomandibular joint engineering: state of art and future persectives. J Craniofac Surg. 2022;33(7):2181–7. 10.1097/SCS.0000000000008771. 10.1097/SCS.0000000000008771 36201705

[61] Rossi F, Tortora C, Paoletta M, Marrapodi MM, Argenziano M, Di Paola A, Pota E, Di Pinto D, Di Martino M, Iolascon G. Osteoporosis in childhood cancer survivors: physiopathology prevention therapy and future perspectives. Cancers (Basel). 2022;14(18):4349. 10.3390/cancers14184349. 10.3390/cancers14184349 PMC9496695 36139510

[62] Franco R, Miranda M, Di Renzo L, De Lorenzo A, Barlattani A, Bollero P. Glanzmann’s thrombastenia: the role of tranexamic acid in oral surgery. Case Rep Dent. 2018;2018:1–4. 10.1155/2018/9370212. 10.1155/2018/9370212 PMC6145161 30254767

[63] Rosa A, Miranda M, Franco R, Guarino MG, Barlattani A Jr, Bollero P. Experimental protocol of dental procedures in patients with hereditary angioedema: the role of anxiety and the use of nitrogen oxide. Oral Implantol (Rome). 2016;9(2):49–53. 10.11138/orl/2016.9.2.049. 10.11138/orl/2016.9.2.049 PMC5159909 28042430

[64] Inchingolo F, Tatullo M, Abenavoli FM, Marrelli M, Inchingolo AD, Gentile M, Inchingolo AM, Dipalma G. Non-syndromic multiple supernumerary teeth in a family unit with a normal karyotype: case report. Int J Med Sci. 2010;378–84. 10.7150/ijms.7.378. 10.7150/ijms.7.378 PMC2974166 21060725

